# Integrating basic research with prevention/intervention to reduce risky substance use among college students

**DOI:** 10.3389/fpsyg.2015.00544

**Published:** 2015-05-07

**Authors:** Danielle M. Dick, Linda C. Hancock

**Affiliations:** ^1^Department of Psychiatry, Virginia Commonwealth University, Richmond, VA, USA; ^2^Department of Psychology, Virginia Commonwealth University, Richmond, VA, USA; ^3^Department of African American Studies, Virginia Commonwealth University, Richmond, VA, USA; ^4^Department of Human and Molecular Genetics, Virginia Commonwealth University, Richmond, VA, USA; ^5^Division of Student Affairs, Wellness Resource Center, Virginia Commonwealth University, Richmond, VA, USA

**Keywords:** spit for science, prevention, intervention, substance use, college students

## Abstract

Too often basic research on etiological processes that contribute to substance use outcomes is disconnected from efforts to develop prevention and intervention programming. Substance use on college campuses is an area of concern where translational efforts that bring together basic scientists and prevention/intervention practitioners have potential for high impact. We describe an effort at a large, public, urban university in the United States to bring together researchers across the campus with expertise in college behavioral health with university administration and health/wellness practitioners to address college student substance use and mental health. The project “Spit for Science” examines how genetic and environmental influences contribute to behavioral health outcomes across the college years. We argue that findings coming out of basic research can be used to develop more tailored prevention and intervention programming that incorporates both biologically and psychosocially influenced risk factors. Examples of personalized programming suggest this may be a fruitful way to advance the field and reduce risky substance use.

## Translational Research in Psychological Science: Obstacles and Opportunities

Translational research has been a priority of the National Institutes of Health for many years (Butler, 2008); however, a chasm remains between basic research and the application of that research to alleviate illness. This gap is often discussed within the realm of medicine with respect to the application of basic, bench-side research to patient treatments (“bench to bedside”). However, it is no less relevant in the field of psychology, where basic psychological scientists often conduct research with little connection to researchers or practitioners involved in the more applied prevention and intervention work that their research informs. Basic researchers, applied researchers, and practitioners often attend their own conferences and publish in their own journals, which hampers by-directional feedback that could be informative for both sides.

In the fall of 2011, we launched a university-wide research project (“Spit for Science”; Dick et al., 2014) focused on genetic and environmental influences on substance use and mental health among college students. This project provided the impetus for an effort to bring together relevant individuals across the university concerned about student substance use. Here we provide an overview of this university initiative and our perspective on how developing connections between basic researchers and applied practitioners can be mutually beneficial. We argue that the university setting is a tremendous opportunity to forge these translational relationships.

## College Student Substance Use: The Need for Improved Approaches

Risky substance use among college students is widespread, with 39% of students reporting that they are binge drinkers (Substance Abuse and Mental Health Services Administration, 2012), and 36% of students reporting illicit drug use in the past year (Kilmer and Geisner, 2013). Nearly half (47%) of all students meet criteria for an alcohol or marijuana use disorder at least once in the first 3 years of college (Caldiera et al., 2009). Substance use is associated with a number of adverse consequences, including decreased academic performance and graduation rates (Kitzrow, 2003), as well as unwanted sexual encounters, legal consequences, assault, injury, suicide, and death (Wechsler et al., 2002; Hingson et al., 2009; Arria et al., 2013). Further, problematic substance use affects the broader university and neighboring communities, contributing to higher rates of sleep and study disruption, property damage, noise complaints, and verbal, physical and sexual violence (Wechsler and Nelson, 2008).

Effectively addressing substance use requires a coordinated approach across the university and its academic and administrative units that views mental and behavioral health as the foundation for student success. College is one of the few times in a person’s life where a single integrated setting encompasses all primary activities, both career-related and social, as well as services related to health, safety, and well-being. This represents a tremendous opportunity to address health and wellness among a segment of the population entering a high-risk developmental phase. Further, by nature of their teaching and research missions, colleges represent an ideal forum from which to develop best-practices for addressing health and wellness. Since many universities possess on their campuses researchers with expertise in relevant areas of study, it is striking that there has been so little systematic research that integrates basic science and intervention research into a unified university approach. There is a need to develop and evaluate programs to improve prevention, identification, and treatment of substance use and mental health problems on college campuses and to translate these findings into policy and practice (Hunt and Eisenerg, 2010).

There is tremendous variability in practices across different campuses, with many using approaches that are sub-optimal (Winters et al., 2011). A survey of colleges examining implementation of the 2002 NIAAA college drinking task force recommendations (http://www.collegedrinkingprevention.gov/) found that the primary approach colleges use to address student alcohol use is education, *a strategy that research has demonstrated is ineffective on its own* (Nelson et al., 2010). Further, only half of surveyed colleges offered empirically supported intervention programs for students who were problem drinkers. Clearly, there is a huge gap between the research basis and translation into best-practices on college campuses.

## “Spit for Science”: A Translational Initiative Focused on College Behavioral Health

At our university, we have considerable research expertise in the area of substance use and mental health. In the fall of 2011, we launched a university-wide research project focused on substance use and mental health. The scientific goal of the project is in-line with other on-going projects of faculty in this area: to understand how genetic and environmental factors impact the development of substance use and mental health problems. However, Spit for Science is unique in that we partnered from the beginning with campus administration and wellness practitioners to create a university-wide initiative aimed at benefitting our students and our local community.

### Methods

All incoming first-time freshmen age 18 or older are invited to complete an on-line survey at the beginning of their fall semester. The survey contains questions about personality and behavior, as well as family, friends, and experiences growing up, and takes approximately 15–30 min to complete. Students receive $10 and a free “Spit for Science” t-shirt for completing the survey. They also have the opportunity to provide a saliva sample for the DNA component (hence the “spit” in Spit for Science), for which they receive another $10. Students have the option of participating in the survey portion of the project and not the DNA component. We do considerable educational outreach about the DNA component and how health outcomes, including substance use and mental health, are a product of our environments and our genetic predispositions. Nearly 70% of eligible freshmen have participated in the project each year, with ∼97% also choosing to participate in the DNA component. The sample is representative of the overall university population in terms of gender and racial/ethnic breakdown. After 4 years of enrolling incoming freshmen, we have over 9,600 students participating in the project. Students are invited to complete a follow-up survey every spring thereafter. Accordingly, the project allows us to understand patterns of substance use and mental health among our students when they start at the university, and the risk and protective factors that impact behavior across their college years (and beyond). For the first cohort, 80% of eligible participants completed the freshman follow-up survey, 59% completed the sophomore follow-up, 53% the junior follow-up, and 45% have completed the senior follow-up (on-going). Students are reminded of spring follow-up surveys via e-mail, mailed information, advertisements around the university and on campus busses, student “recruiters” passing out flyers and manning information tables, dorm visits and educational events. We are currently implementing a number of additional initiatives intended to bolster retention, including feeding back study results to students and enhancing our use of social media to provide a more interactive on-going connection between students and the project.

All participants are enrolled in a registry, allowing faculty across the university to work with the data and focus on areas of behavioral health specific to their expertise. The registry also allows for selection of individuals with particular characteristics (e.g., phenotypes or genotypes of interest) for more intensive “spin-off” studies. In addition to the primary analyses focused on alcohol use, there are currently additional projects underway examining nicotine use, depression, anxiety, eating disorders, trauma, sleep, physical exercise, parenting, peer and romantic relationships, family history, and more. Much of the success of the project can be attributed to a highly collaborative group of faculty researchers and prevention/intervention staff, as well as university administrators who supported the project and helped us navigate the logistical hurdles involved in launching a cross-campus initiative of this magnitude. Additional details about the data collection and the groundwork that went into launching this university-wide research project can be found in Dick et al. (2014).

Although the project is on-going, basic researchers and prevention practitioners have already started implementing interventions based on survey findings, including a large campus-wide social norms marketing primary prevention intervention. This mass media simultaneously maximizes study participation by raising awareness about the project and acts strategically as an intervention to provide normative results and health information in a motivational style to students. Monthly 1100 posters are placed in bathrooms across campus (examples can be accessed through www.thewell.vcu.edu). Research shows that half the campus are “high-readers” reading over half to all of the publication multiple times.

### Initial Results

Rates of substance use in the Spit for Science sample are consistent with other large studies of college-age populations. For example, in Spit for Science 72% of participants report having tried alcohol, compared to 71% in the Monitoring the Future (MTF) post 12th grade assessment (Johnston et al., 2010); the prevalence of marijuana use is 41% in Spit for Science and 44% in MTF (additional details in Dick et al., 2014). In initial analyses we examined patterns of substance use and potential changes in those patterns across the transitional first year of college by conducting a latent class analysis of substance use as reported at the beginning of the fall freshman semester, and midway through the spring semester (Cho et al., under review). At both timepoints, three classes of individuals emerged: (1) polysubstance users, with relatively higher levels of use of alcohol, tobacco, cannabis, and other illicit drugs (2) alcohol, tobacco, cannabis users; and (3) low substance users, with overall low levels of use, with alcohol use being the most common. Interestingly, very few participants transitioned in their substance use class across the first year of college. This suggests that college students largely maintain the patterns of substance use *with which they enter college*. This finding is consistent with a previous study that reported pre-college heavy drinking to be the strongest predictor of heavy drinking in the first semester (Sher and Rutledge, 2007). These studies suggest that high-risk users have previously established substance use patterns prior to college, a finding that has important implications for early intervention efforts: *we know who is most at risk from the time they step onto our campus*. This underscores the importance of implementing effective, empirically supported prevention efforts that go beyond “one-size-fits-all.”

## Integrating Developmental Epidemiology with Prevention/Intervention

The current “gold standard” for reducing risky alcohol use among college students is the use of brief motivational feedback interventions (Larimer and Cronce, 2002; Lee et al., 2010), which have demonstrated efficacy delivered via in-person and web-based platforms (Chiauzzi et al., 2005; Carey et al., 2009; Hustad et al., 2010). These programs generally combine elements of cognitive-behavioral skill training, and personalized feedback in a motivational interviewing style. They provide students with information about how their drinking compares to campus norms. They also help students see possible consequences associated with excessive alcohol use such as impact on academic performance and career goals, and they empower students to undertake new strategies to monitor their drinking, set limits, and reduce risk. They have been adopted for both universal prevention programming intended for all college students, and targeted programming for mandated students (Barnett et al., 2004; Bosari and Carey, 2005; White et al., 2006; Hustad et al., 2010).

What is striking about this literature is that there is little integration with the body of research on pathways of risk for alcohol problems. Individuals use and abuse alcohol for different reasons (Heinz et al., 2003), and the development of alcohol-related problems is often discussed within the context of multiple pathways. In a study we conducted on the association between early childhood temperamental factors and adolescent alcohol use using data from >12,000 individuals followed from birth, we found two distinct temperamental/behavioral patterns evident before age 5 that predicted mid-adolescent alcohol use: (1) children who were rated as having consistent emotional and conduct problems and (2) children who were rated as consistently sociable both had elevated rates of alcohol use in adolescence (Dick et al., 2013). An externalizing pathway, characterized by behavioral undercontrol, sensation-seeking, impulsivity, and antisocial behavior, has been robustly associated with alcohol problems (Zucker, 2008), and there is more modest evidence for a risk pathway characterized by internalizing symptomatology (Zucker, 2008; Hussong et al., 2011). Further, individuals with alcoholism also show considerable heterogeneity, with a common distinction being alcoholics who have antisocial/externalizing traits and alcoholics who have anxious/depressive comorbid features (Cloninger et al., 1981; Babor et al., 1992). These literatures all indicate that individuals who misuse alcohol are a heterogeneous group. Yet our current prevention/intervention strategies often apply a one-size-fits-all strategy that focuses almost entirely on alcohol use and not on the various factors that (differentially) affect risk.

Recently, a literature has begun to emerge that focuses on prevention programming tailored to individual risk profiles. Conrod et al. (2013) developed a school-based alcohol prevention program that targets personality risk profiles: anxiety sensitivity, hopelessness, impulsivity, and sensation-seeking, and shows robust effects on reducing adolescent drinking behavior (Conrod et al., 2013; O’Leary-Barret et al., 2013). Schuckit et al. (2009) developed a tailored intervention focused on low level of response to alcohol, a known biological risk factor reflecting a need for larger amounts of alcohol to experience effects, that has been robustly associated with higher alcohol intake and increased risk for the development of alcohol-related problems (Schuckit et al., 2009). Level of response can be assessed using a brief set of self-report questions that ask an individual to report on the number of drinks it took them to experience various effects of alcohol (slurred speech, stumbling, etc.) when they first began drinking. In a pilot study of college freshmen, individuals who reported a low level of response to alcohol and who were assigned to a prevention program structured around how a low physiological response affects heavy drinking, showed greater decreases in alcohol use as compared to individuals with a low level of response who were assigned to a standard prevention program that covered the same information, but not in the context of a low level of response framework. Individuals who did not have a low level of response to alcohol did better in the standard prevention program (Schuckit et al., 2012). We assessed level of response in the Spit for Science survey and used these data to invite a subset of students to participate in an intervention study designed to replicate the Schuckit et al. (2012) finding. We too found modest support for individuals with a low level of response showing decreased alcohol use in the level of response prevention program compared to the non-tailored program, particularly for high risk drinking practices such as maximum number of drinks in a day (Savage et al., unpublished). Interestingly, we found that the individuals with a low level of response (i.e., those most at risk) showed greater decreases in alcohol use to both prevention programs compared to individuals with a high level of response. These findings are consistent with results from another line of prevention work by Brody et al. (2009) in which children who were characterized as at risk based on their genetic profiles showed the greatest benefit from prevention programming aimed at reducing adolescent alcohol use (Brody et al., 2009). Though risk was characterized in different ways across these studies (physiological response versus measured genotypic risk), and different prevention programs were implemented with different populations, both studies found that those at greatest risk benefited most from prevention programming.

We believe that there is great potential to integrate these literatures to develop more targeted prevention and intervention programming for college students that focuses on individual risk factors. Although brief motivational feedback interventions for college student substance use have demonstrated efficacy, the effects associated with these programs are modest (Walters and Neighbors, 2005; Rooke et al., 2010) and risky alcohol use remains widespread on college campuses (Timberlake et al., 2007; Martinez et al., 2008; Wechsler and Nelson, 2008; Kilmer and Geisner, 2013). Personalized feedback is thought to be one of the critical elements contributing to the effectiveness of extant college prevention programs (Walters and Neighbors, 2005). Integrating findings on risk factors associated with alcohol use would make it possible to provide feedback based on more comprehensive risk profiles that extend beyond current patterns of alcohol use. We are currently working on using technology-based platforms to provide individual feedback across multiple dimensions (e.g., level of response, personality, externalizing and internalizing characteristics) in order to test whether enhanced personalized feedback improves prevention/intervention outcomes. The ability to use technology to personalize feedback also obviates the need to group individuals into subtypes (e.g., high/low responders, impulsive, anxious, etc.) as each individual can have their own personalized risk profile. The emerging literature on the enhanced effectiveness of tailored prevention programming suggests this may be a fruitful way forward.

## Where Does Genetics Fit In?

As part of the Spit for Science project we collect DNA. We are clear with our students that the DNA will be used for basic research purposes only, to identify genes that are involved in why some people are more likely to develop problems associated with substance use and mental health than others, and to understand how the environment can moderate risk for those who are genetically predisposed. We are explicit that students will not receive feedback about their risk. The science simply is not at a point where that information is useful. This is illustrated by analyses conducted by a graduate student in my (DD) lab who completed a Ph.D. in genetics and genetic counseling in which she evaluated the predictive ability of known genes associated with alcohol dependence and found that at our current level of knowledge they predict no better than chance (Yan et al., 2013). Family history remained the most robust predictor. Polygenic risk scores combining information across the genome currently predict only ∼1–3% of the variance in alcohol-related outcomes (Salvatore et al., 2014). However, as our gene finding efforts advance, the percent of variance explained by known genetic risk factors is likely to grow, as evidenced in other areas where the amount of variance explained by polygenic risk scores has become non-trivial (e. g., 60% for type- I diabetes; 10% for height; Visscher et al., 2012). Identifying these risk genes has required huge samples (180,000 individuals for height! Lango Allen et al., 2010), and efforts to grow large-scale collaborations are underway for substance dependence. However, genetics will always be just part of the puzzle, with substance use disorders having a heritability in the range of 50–70% (Verhulst et al., 2015). Further, it remains unclear how individuals would use personalized genetic information. The FDA’s current ban on direct to consumer genetic testing (U.S. Food and Drug Administration, 2013), and the considerable controversy that surrounded the University of California Berkley’s provision of genetic results to its students as an academic exercise to stimulate discussion about personalized genetic feedback (Gruber, n.d.), underscore the uncertainty surrounding how personalized genetic information may be integrated into efforts aimed at prevention/intervention and improving human health.

However, these challenges are not insurmountable, and there are ways that genetically informative information can be useful in the interim, even before we have identified genes and have a clearer sense of how to use this information. We know that there are no genes “for” substance use or mental health outcomes anyway. Rather, genetic factors impact distal clinical outcomes through intermediary traits and pathways. For example, in the area of substance use, genetic factors that impact risk for the development of substance use problems likely act through intermediary mechanisms such as personality and physiological response to alcohol. Twin studies and molecular genetic studies have demonstrated that genetic influences that impact adult alcohol use outcomes can manifest as conduct problems earlier in development, and also impact other indices of behavioral disinhibition, such as sensation-seeking and novelty seeking (Young et al., 2000; Krueger et al., 2002; Dick et al., 2009; Aliev et al., 2015). Accordingly, we can use these more proximal traits that are part of the pathway of risk influenced by the underlying predisposition for personalized feedback, which will allow us to further study how the provision of individual risk information influences the effectiveness of prevention/intervention programming. We think there is great potential for basic researchers to work with prevention/intervention practitioners to develop interactive feedback programming that provides students with more comprehensive information about their profile of risk (potentially including biologically and psychosocially influenced factors), in order to give them insight into factors that affect their substance use and mental health.

## Conclusion

University settings provide great potential for translational efforts that bring together basic scientists, prevention/intervention practitioners, and university administrators. Substance use and behavioral health concerns on college campuses are areas of high impact for the university community where these translational efforts hold great promise. The effectiveness of new, personalized prevention programming suggests that integrating etiological information into prevention and intervention efforts may provide new and innovative ways to address challenging, common problems among young adults. Basic scientists can provide information about the pathways of risk involved in behavioral health challenges, while benefitting from collaborative discussions with practitioners that can inform the research. By teaming up with researchers, practitioners can benefit from the knowledge of local research expertise, and enhance their ability to evaluate program effectiveness. Administrators who have access to data showing the connection between substance use and student success outcomes on their campus may have a stronger impetus to fund both the research and intervention programs focused on substance use and mental health at the university. In the end, more collaborative translational research interventions hold great promise for all involved.

## Author Contributions

Both authors contributed equally to the creation of this work and approve this version to be published.

### Conflict of Interest Statement

The authors declare that the research was conducted in the absence of any commercial or financial relationships that could be construed as a potential conflict of interest.

## References

[B1] Aliev F. Wetherill L. Bierut L. Bucholz K. Edenberg H. Foroud T. (2015). Genes associated with alcohol outcomes show enrichment of effects with broad externalizing and impulsivity phenotypes in an independent sample. J. Stud. Alcohol. Drugs 76, 38–46. 10.15288/jsad.2015.76.38 25486392 PMC4263779

[B2] Arria A. M. Wilcox H. C. Caldeira K. M. Vincent K. B. Garnier-Kykstra L. M. O’grady K. E. (2013). Dispelling the myth of “smart drugs”: cannabis and alcohol use problems predict nonmedical use of prescription stimulants for studying. Addict. Behav. 38, 1643–1650. 10.1016/j.addbeh.2012.10.002 23254212 PMC3558594

[B3] Babor T. F. Hofmann M. Delboca F. K. Hesselbrock V. Meyer R. E. Dolinsky Z. S. (1992). Types of alcoholics, I. Evidence for an empirically derived typology based on indicators of vulnerability and severity. Arch. Gen. Psychiatry 49, 599. 10.1001/archpsyc.1992.01820080007002 1637250

[B4] Barnett N. P. Tevyaw T. O. Fromme K. Bosari B. Carey K. B. Corbin W. R. (2004). Brief alcohol interventions with mandated or adjudicated college students. Alcohol. Clin. Exp. Res. 28, 966–975. 10.1097/01.ALC.0000128231.97817.C7 15218881 PMC2726616

[B5] Bosari B. Carey K. B. (2005). Two brief alcohol interventions for mandated college students. Psychol. Addict. Behav. 19, 296–302. 10.1037/0893-164X.19.3.296 16187809 PMC2663045

[B6] Brody G. H. Beach S. R. H. Philibert R. A. Chen Y. C. Murry V. M. (2009). Prevention effects moderate the association of the 5HTTLPR and youth risk behavior initiation: gene × environment hypotheses tested via randomized prevention design. Child Dev. 80, 645–661. 10.1111/j.1467-8624.2009.01288.x 19489894

[B7] Butler D. (2008). Translational research: crossing the valley of death. Nature 453, 840–842. 10.1038/453840a 18548043

[B8] Caldiera K. Kasperski S. J. Sharma E. Vincent K. B. O’grady K. E. Wish E. D. (2009). College students rarely seek help despire serious substance use problems. J. Subst. Abuse Treat. 37, 368–378. 10.1016/j.jsat.2009.04.005 19553064 PMC2783958

[B9] Carey K. B. Henson J. M. Carey M. P. Maisto S. A. (2009). Computer versus in-person intervention for students violating campus alcohol policy. J. Consult. Clin. Psychol. 77, 74–87. 10.1037/a0014281 19170455 PMC2657221

[B10] Chiauzzi E. Green T. C. Lord S. Thum C. Goldstein M. (2005). My student body: a high-risk drinking prevention web site for college students. J. Am. Coll. Health 53, 263–274. 10.3200/JACH.53.6.263-274 15900990 PMC1885481

[B11] Cloninger C. R. Bohman M. Sigvardsson S. (1981). Inheritance of alcohol abuse. Cross-fostering analysis of adopted men. Arch. Gen. Psychiatry 38, 861. 10.1001/archpsyc.1981.01780330019001 7259422

[B12] Conrod P. J. O’leary-Barrett M. Newton N. C. Topper L. Castellanos-Ryan N. Mackie C. (2013). Effectiveness of a selective, personality-targeted prevention program for adolescent alcohol use and misuse. JAMA Psychiatry 70, 334–342. 10.1001/jamapsychiatry.2013.651 23344135

[B13] Dick D. M. Aliev F. Latendresse S. J. Hickman M. Heron J. Macleod J. (2013). Adolescent alcohol use is predicted by childhood temperament factors before age 5, with mediation through personality and peers. Alcohol. Clin. Exp. Res. 37, 2108–2117. 10.1111/acer.12206 23841856 PMC3823677

[B14] Dick D. M. Latendresse S. J. Lansford J. E. Budde J. P. Goate A. Dodge K. A. (2009). Role of GABRA2 in trajectories of externalizing behavior across development and evidence of moderation by parental monitoring. Arch. Gen. Psychiatry 66, 649–657. 10.1001/archgenpsychiatry.2009.48 19487630 PMC2750080

[B15] Dick D. M. Nasim A. Edwards A. C. Salvatore J. E. Cho S. B. Adkins A. (2014). Spit for science: launching a longitudinal study of genetic and environmental influences on substance use and emotional health at a large US university. Front. Genet. 5:47. 10.3389/fgene.2014.00047 24639683 PMC3944794

[B16] Gruber J. (n.d.). UC Berkeley Adopts Controversial Genetic Testing Program. Genewatch [Online] Available at: http://www.councilforresponsiblegenetics.org/genewatch/GeneWatchPage.aspx?pageId=269 [accessed March 9, 2015].

[B17] Heinz A. Lber S. Georgi A. Wrase J. Hermann D. Rey E.-R. (2003). Reward craving and withdrawal relief craving: assessment of different motivational pathways to alcohol intake. Alcohol. Alcohol. 38, 35–39. 10.1093/alcalc/agg005 12554605

[B18] Hingson R. W. Zha W. Weitzman E. R. (2009). Magnitude of and trends in alcohol-related mortality and morbidity among U.S. college students ages 18–24, 1998–2005. J. Stud. Alcohol. Drugs Suppl. 16, 12–20. 10.15288/jsads.2009.s16.12 19538908 PMC2701090

[B19] Hunt J. Eisenerg D. (2010). Mental health problems and help-seeking behavior among college students. J. Adolesc. Health 46, 3–10. 10.1016/j.jadohealth.2009.08.008 20123251

[B20] Hussong A. M. Jones D. J. Stein G. L. Baucom D. H. Boeding S. (2011). An internalizing pathway to alcohol use and disorder. Psychol. Addict. Behav. 25, 390–404. 10.1037/a0024519 21823762 PMC3178003

[B21] Hustad J. Barnett N. P. Borsari B. Jackson K. M. (2010). Web-based alcohol prevention for incoming college students: a randomized controlled trial. Addict. Behav. 35, 183–189. 10.1016/j.addbeh.2009.10.012 19900763 PMC2815230

[B22] Johnston L. D. Bachman J. G. O’malley P. M. (2010). Monitoring the Future: Questionnaire Responses From the Nation’s High School Seniors, 2009. Ann Arbor, MI: Institute for social research.

[B23] Kilmer J. R. Geisner I. M. (2013). “Substance use and mental health issues on the college campus,” in Principles of Addiction: Comprehensive Addictive Behaviors and Disorders, ed. Miller P. (London: Academic Press), 597–604.

[B24] Kitzrow M. A. (2003). The mental health needs of today’s college students: challenges and recommendations. NASPA 41, 167–181 10.2202/1949-6605.1310

[B25] Krueger R. F. Hicks B. M. Patrick C. J. Carlson S. R. Iacono W. G. Mcgue M. (2002). Etiologic connections among substance dependence, antisocial behavior, and personality: modeling the externalizing spectrum. J. Abnorm. Psychol. 111, 411–424. 10.1037/0021-843X.111.3.411 12150417

[B26] Lango Allen H. Estrada K. Lettre G. Berndt S. I. Weedon M. N. Rivadeneira F. (2010). Hundreds of variants clustered in genomic loci and biological pathways affect human height. Nature 467, 832–838. 10.1038/nature09410 20881960 PMC2955183

[B27] Larimer M. E. Cronce J. M. (2002). Identification, prevention and treatment: a review of individual-focuses strategies to reduce problematic alcohol consumption by college students. J. Stud. Alcohol. 32, 148–163. 10.15288/jsas.2002.s14.148 12022721

[B28] Lee C. M. Neighbors C. Kilmer J. R. Larimer M. E. (2010). A brief, web-based personalized feedback selective intervention for college student marijuana use: a randomized clinical trial. Psychol. Addict. Behav. 24, 265–273. 10.1037/a0018859 20565152 PMC2891541

[B29] Martinez J. A. Sher K. Wood P. K. (2008). Is heavy drinking really associated with attrition from college? The alcohol-attrition paradox. Psychol. Addict. Behav. 22, 450–456. 10.1037/0893-164X.22.3.450 18778140 PMC2673793

[B30] Nelson T. F. Toomey T. Lenk K. Erickson D. Winters K. C. (2010). Implementation of NIAAA college drinking task force recommendations: how are colleges doing 6 years later? Alcohol. Clin. Exp. Res. 34, 1687–1693. 10.1111/j.1530-0277.2010.01268.x 20626728

[B31] O’Leary-Barret M. Topper L. Al-Khudhairy N. Pihl R. O. Castellanos-Ryan N. Mackie C. (2013). Two-year impact of personality-targeted, teacher-delivered interventions on youth internalizing and externalizing problems: a cluster-randomized trial. J. Am. Acad. Child Adolesc. Psychiatry 52, 911–920. 10.1016/j.jaac.2013.05.020 23972693

[B32] Rooke S. Thorsteinsson E. Karpin A. Copeland J. Allsop D. (2010). Computer-delivered interventions for alcohol and tobacco use: a meta-analysis. Addiction 105, 1381–1390. 10.1111/j.1360-0443.2010.02975.x 20528806

[B33] Salvatore J. Aliev F. Edwards A. Evans D. Macleod J. Hickman M. (2014). Polygenic scores predict alcohol problems in an independent sample and show moderation by the environment. Genes 5, 330–346. 10.3390/genes5020330 24727307 PMC4094936

[B34] Schuckit M. A. Smith T. L. Danko G. P. Trim R. Bucholz K. K. Edenberg H. J. (2009). An evaluation of the full level of response to alcohol model of heavy drinking and problems in COGA offspring. J. Stud. Alcohol. Drugs 70, 436–445. 10.15288/jsad.2009.70.436 19371495 PMC2670749

[B35] Schuckit M. Kalmijn J. A. Smith T. L. Saunders G. Fromme K. (2012). Structuring a college alcohol prevention program on the low level of response to alcohol model: a pilot study. Alcohol. Clin. Exp. Res. 36, 1244–1252. 10.1111/j.1530-0277.2011.01723.x 22309202

[B36] Sher K. Rutledge P. C. (2007). Heavy drinking across the transition to college: predicting first-semester heavy drinking from precollege variables. Addict. Behav. 32, 819–335. 10.1016/j.addbeh.2006.06.024 16860940 PMC2674234

[B37] Substance Abuse and Mental Health Services Administration. (2012). “Results from the 2011 National Survey on Drug Use and Health: Summary of National Findings,” in NSDUH Series H-44, HHS Publication No. (SMA) 12-4713, (Rockville, MD: Substance Abuse and Mental Health Services Administration).

[B38] Timberlake D. Hopfer C. J. Rhee S. H. Friedman N. P. Haberstick B. Lessem J. M. (2007). College attendance and its effect on drinking behaviors in a longitudinal study of adolescents. Alcohol. Clin. Exp. Res. 31, 1020–1030. 10.1111/j.1530-0277.2007.00383.x 17403064

[B39] U.S. Food and Drug Administration. (2013). Inspections, Compliance, Enforcement, and Criminal Investigations. U.S. Department of Health and Human Services [Online] Available at: http://www.fda.gov/ICECI/EnforcementActions/WarningLetters/2013/ucm376296.htm [accessed March 9, 2015].

[B40] Verhulst B. Neale M. C. Kendler K. S. (2015). The heritability of alcohol use disorders: a meta-analysis of twin and adoption studies. Psychol. Med. 45, 1061–1072. 10.1017/S0033291714002165 25171596 PMC4345133

[B41] Visscher P. M. Brown M. A. Mccarthy M. I. Yang J. (2012). Five years of GWAS discovery. Am. J. Hum. Genet. 90, 7–24. 10.1016/j.ajhg.2011.11.029 22243964 PMC3257326

[B42] Walters S. T. Neighbors C. (2005). Feedback interventions for college alcohol misuse: what, why and for whom? Addict. Behav. 30, 1168–1182. 10.1016/j.addbeh.2004.12.005 15925126 PMC2459313

[B43] Wechsler H. Lee J. E. Kuo M. Seibring M. Nelson T. F. Lee H. (2002). Trends in college binge drinking during a period of increased prevention efforts: findings form 4 Harvard School of Public Health College Alcohol Study surveys: 1993-2001. J. Am. Coll. Health 50, 203–217. 10.1080/07448480209595713 11990979

[B44] Wechsler H. Nelson T. (2008). What we have learned from the Harvard School of Public Health College Alcohol Study: focusing attention on college student alcohol consumption and the environmental conditions that promote it. J. Stud. Alcohol. Drugs 69, 481–490. 10.15288/jsad.2008.69.481 18612562

[B45] White A. M. Kraus C. L. Swartzwelder H. S. (2006). Many college freshmen drink at levels far beyond the binge threshold. Alcohol. Clin. Exp. Res. 30, 1006–1010. 10.1111/j.1530-0277.2006.00122.x 16737459

[B46] Winters K. C. Toomey T. Nelson T. F. Erickson D. Lenk K. Miazga M. (2011). Screening for alcohol problems among 4-year colleges and universities. J. Am. Coll. Health 59, 350–357. 10.1080/07448481.2010.509380 21500052 PMC3086775

[B47] Yan J. Aliev F. Webb B. T. Kendler K. S. Edenberg H. J. Agrawal A. (2013). Using genetic information from candidate gene and genome wide association studies in risk prediction for alcohol dependence. Addict. Biol. 18, 708–721. 10.1111/adb.12035 23362995 PMC3664249

[B48] Young S. E. Stallings M. C. Corley R. P. Krauter K. S. Hewitt J. K. (2000). Genetic and environmental influences on behavioral disinhibition. Am. J. Med. Genet. 96, 684–695. 10.1002/1096-8628(20001009)96:5<684::AID-AJMG16>3.0.CO;2-G 11054778

[B49] Zucker R. A. (2008). Anticipating problem alcohol use developmentally from childhood into middle adulthood: what have we learned. Addiction 103, 100–108. 10.1111/j.1360-0443.2008.02179.x 18426543 PMC2593849

